# Cortical organization of inhibition-related functions and modulation by psychopathology

**DOI:** 10.3389/fnhum.2013.00271

**Published:** 2013-06-13

**Authors:** Stacie L. Warren, Laura D. Crocker, Jeffery M. Spielberg, Anna S. Engels, Marie T. Banich, Bradley P. Sutton, Gregory A. Miller, Wendy Heller

**Affiliations:** ^1^Department of Psychology, University of Illinois at Urbana-ChampaignChampaign, IL, USA; ^2^Department of Mental Health, St. Louis VA Medical CenterSt. Louis, MO, USA; ^3^School of Public Health, University of California, BerkeleyBerkeley, CA, USA; ^4^Department of Psychology, Pennsylvania State UniversityUniversity Park, PA, USA; ^5^Department of Psychology, University of Colorado at BoulderBoulder, CO, USA; ^6^Department of Bioengineering, University of Illinois at Urbana-ChampaignUrbana, IL, USA; ^7^Department of Psychology, University of DelawareNewark, DE, USA; ^8^Department of Psychology, University of KonstanzKonstanz, Germany

**Keywords:** inhibition, anxiety, depression, DLPFC, attentional control

## Abstract

Individual differences in inhibition-related functions have been implicated as risk factors for a broad range of psychopathology, including anxiety and depression. Delineating neural mechanisms of distinct inhibition-related functions may clarify their role in the development and maintenance of psychopathology. The present study tested the hypothesis that activity in common and distinct brain regions would be associated with an ecologically sensitive, self-report measure of inhibition and a laboratory performance measure of prepotent response inhibition. Results indicated that sub-regions of DLPFC distinguished measures of inhibition, whereas left inferior frontal gyrus and bilateral inferior parietal cortex were associated with both types of inhibition. Additionally, co-occurring anxiety and depression modulated neural activity in select brain regions associated with response inhibition. Results imply that specific combinations of anxiety and depression dimensions are associated with failure to implement top-down attentional control as reflected in inefficient recruitment of posterior DLPFC and increased activation in regions associated with threat (MTG) and worry (BA10). Present findings elucidate possible neural mechanisms of interference that could help explain executive control deficits in psychopathology.

## Introduction

Despite a lack of consensus on how best to define executive function (EF), neuropsychological and neuroimaging (Collette et al., 2005) research indicates that EF may be usefully characterized as a collection of correlated yet dissociable processes: inhibition, set shifting, and working memory updating (e.g., Miyake et al., 2000). Inhibition-related processes in particular are considered to be critical for top-down cognitive control and its translation to real-word, everyday behavior, including self-regulation and emotion regulation (Zelazo and Cunningham, 2007). Further, inhibition-related functions are essential for efficient working memory function, limiting access to and removing information that is no longer necessary (Friedman and Miyake, 2004). Cognitive disruptions in these processes are a prominent source of distress and impairment and have been implicated in anxiety and depression (Eysenck et al., 2007; Levin et al., 2007; Joormann and Gotlib, 2010; Snyder, 2013; Warren et al., under review). To the degree that the experience of negative mood states and negative life events activates mood-congruent representations in working memory (Siemer, 2005), identifying specific inhibition-related dysfunctions associated with anxiety and depression could lead to relatively specific targets for intervention.

Not only do inhibition-related processes contribute to aspects of daily life, they play a critical role in psychopathology, as deficits in these processes have been implicated in the affective and cognitive symptoms of anxiety and depression. In particular, intrusive thoughts such as worry and rumination are hallmark characteristics of anxiety and depression, respectively, and several researchers have suggested that these symptoms are a result of impaired inhibition (Hertel, 1997; Eysenck et al., 2007; Joormann, 2010; see Anticevic et al., 2012, and Fox et al., 2012, for potential contributions of neural networks to psychopathology symptoms). Anxiety has been associated with broad impairments in attentional control, including increased distractibility and impaired processing efficiency (e.g., resource utilization) as opposed to performance effectiveness (e.g., percentage of correct responses; Eysenck et al., 2007; Eysenck and Derakshan, 2011). Research in depression has repeatedly demonstrated problems with attention, memory, and problem-solving abilities (Yee and Miller, 1994; Weiland-Fiedler et al., 2004; Levin et al., 2007; Warren et al., 2008), and impaired inhibition is hypothesized to facilitate these cognitive disruptions via effects on working memory (e.g., Joormann and Gotlib, 2010). Thus, making an explicit link among individual differences in specific inhibition-related functions and dimensions of anxiety and depression is important for understanding the intricate relationship between affective experiences and cognitive control.

Colloquially, the term inhibition is used with respect to control of behaviors in everyday life (e.g., distraction, impulsivity), although the contribution of specific inhibition-related functions is not well understood. Notably, most formal tests of EF were developed and administered in understandably artificial environments (e.g., laboratory or controlled testing environment). Although research is advancing in determining the cognitive processes that these formal tests of EF actually measure (e.g., Miyake et al., 2000), the degree to which activities of daily life require these same processes is unclear (Burgess et al., 2009). The present study sought to identify empirically specific neural mechanisms implementing the type of inhibition that has been demonstrated clearly in a laboratory setting, (e.g., prepotent response inhibition) as well as behavioral inhibition measured in everyday life. Given that impaired inhibition-related functions have been implicated as risk factors for a broad range of psychopathology, it is important that the nature of inhibition-related processes be specified.

Individual differences in specific inhibition-related functions at the level of neural mechanisms might be more strongly tied to the development and maintenance of psychopathology than the broader construct of inhibition as a whole. Neuroimaging studies exploring inhibition have demonstrated the involvement of various regions, including dorsolateral prefrontal cortex (DLPFC), inferior frontal gyrus (IFG), and anterior cingulate cortex (ACC), although lesion studies implicate right IFG in particular (see Aron et al., 2004, for a review). DLPFC, ACC, and IFG appear to facilitate task performance in inhibition paradigms. However, it is likely that inhibition co-exists with other cognitive functions required by these tasks (e.g., updating, shifting), making it difficult to determine which brain regions are involved in the implementation of specific inhibition processes. DLFPC is associated with top-down attentional control (e.g., Dosenbach et al., 2008), maintaining goals, and updating information (e.g., Wager and Smith, 2003), whereas ACC is involved in detecting response conflict and monitoring performance (Nelson et al., 2003; Banich et al., 2009). IFG is activated when an individual needs to resolve interference among potentially conflicting attributes of stimuli (Nelson et al., 2003; for review of left IFG, see Jonides and Nee, 2006) and may function to inhibit incorrect responses (Aron et al., 2004). Further, IFG appears to play a more general role in responding to salient, task-related cues as part of an EF network (Hampshire et al., 2010).

Although there is some support for inhibitory dysfunction in both anxiety and depression, the literature to date is mixed (Derakshan and Eysenck, 2009; Snyder, 2013; Snyder et al., under review). Several methodological and conceptual issues could account for discrepant results. Cognitive tasks that are commonly employed often each rely on multiple aspects of EF that might be impaired in psychopathology, making it difficult to draw firm conclusions about the presence of inhibition-related deficits specifically (Henry and Crawford, 2005). In addition, the concept of “inhibition” is broad, and tasks that are assumed to measure inhibition vary in their definition and implementation, making it difficult to ascertain the nature of the function measured. Finally, evidence suggests that co-occurring disorders may have both additive and interactive effects on brain activity and EF (Keller et al., 2000; Moritz et al., 2001; Basso et al., 2007; Engels et al., 2010; Herrington et al., 2010) as well as on clinical outcomes (e.g., Emmanuel et al., 1998). Yet many studies fail to assess or control comorbidity, making it difficult to parse the effects of specific dimensions of anxiety and depression on inhibition impairments and related brain activity.

Depression is distinguishable from two types of anxiety, anxious apprehension and anxious arousal (Heller et al., 1997; Nitschke et al., 1999, 2001). Anxious apprehension is characterized by worry and verbal rumination (Andrews and Borkovec, 1988; Barlow, 1991, 2002), whereas anxious arousal is characterized by somatic tension and sympathetic hyperarousal (Watson et al., 1995a,b). In contrast, depression is characterized by decreased responsivity to pleasurable stimuli (i.e., anhedonia; APA, 2000) and low positive affect (Watson et al., 1995a).

Hemodynamic neuroimaging studies of anxiety and depression have identified abnormal function in regions associated with inhibition-related processes, including prefrontal cortex (particularly DLPFC and IFG), ACC, and areas within parietal cortex (Mayberg, 1997; Mayberg et al., 1999; Rogers et al., 2004; Pizzagalli et al., 2006; Engels et al., 2007, 2010; Herrington et al., 2010). Further, when distinctions between depression and types of anxiety are taken into account, distinct patterns of neural activity emerge. For example, Engels et al. (2007, 2010) demonstrated that anxious apprehension is associated with increased left IFG (Broca's area) activity, whereas anxious arousal is associated with increased right inferior temporal gyrus (ITG) activity. Herrington et al. (2010; see also Miller et al., 2013) demonstrated that depression is associated with rightward lateralization of DLPFC activity. Given that individual differences in inhibition-related functions have been implicated as risk factors for anxiety and depression, a second goal of the present study is to examine how these dimensions of psychopathology (anxious apprehension, anxious arousal, and anhedonic depression) modulate neural mechanisms supporting specific inhibition-related functions. Understanding these relationships could contribute to an account of psychological or neural mechanisms involved in the development and maintenance of symptoms of psychopathology, as well as inform current and potential methods of treatment targeting the cognitive biases and impairments associated with anxiety and depression.

Based on the review above, it was hypothesized that regions involved in a frontal-parietal network supporting inhibition-related processes will be associated with both self-reported behavioral inhibition in everyday life and prepotent response inhibition. In addition, it was anticipated that distinct neural mechanisms would be associated with the two aspects of inhibition under investigation. It was hypothesized that Stroop reaction time (RT) interference, a measure of prepotent response inhibition that likely reflects greater active suppression than self-reported inhibition in everyday life, would be associated with DLPFC, ACC, and IFG activity. These regions have been implicated in implementing cognitive control, as well as response inhibition (Banich et al., 2000; Milham and Banich, 2005; Banich, 2009). In particular, it was anticipated that RT interference would be associated with posterior DLPFC activity, as this region is considered to be critically involved in performance on the Stroop task, in part by biasing other brain regions toward processing task-relevant information (e.g., color of the ink) and away from task-irrelevant information (e.g., reading the color word). Thus, posterior DLPFC is thought to be particularly involved in implementing resistance to a dominant response. In contrast, it was hypothesized that self-reported behavioral inhibition would be associated with mid-DLPFC activity, as this region is implicated in multitasking functions and responding to context (Crocker et al., 2012), as well as maintaining task-relevant information (Kane and Engle, 2002; Banich, 2009). Thus, mid-DLPFC is associated with resisting distraction. Further, given mid-DLPFC's role in maintaining task-relevant information and resisting distraction, it was anticipated that worse self-reported inhibition (e.g., impulsivity, distractibility) would be associated with increased activity in this area. Given that response-inhibition paradigms have dominated much of the inhibition neuroimaging literature, it is unknown whether self-reported inhibition as measured in everyday life will elicit IFG and ACC activity. To the degree that self-reported inhibition relies on stopping behavioral responses, it is likely to be associated with IFG activation. A correlation with ACC may be less likely, as this region is recruited during tasks that generate conflicting, response-related representations, such as the incongruent condition of a Stroop task (“RED” printed in blue ink; Banich, 2009).

Further, given empirical support from hemodynamic neuroimaging studies that have properly accounted for comorbidity between depression and anxiety or comorbidity among anxiety types (Engels et al., 2007, 2010; Herrington et al., 2010), it was hypothesized that depression and anxiety would be associated with opposing effects on inhibition-related brain activity. For both prepotent response inhibition and self-reported inhibition in everyday life, it was anticipated that depression would be associated with decreased left DLPFC and ACC activity. It was also hypothesized that depression would be associated with decreased posterior DLPFC response inhibition activity, as previous work has shown hypoactivation in this area (e.g., Herrington et al., 2010). In contrast, anxiety should be accompanied by greater activation in brain areas associated with attentional control in distracting conditions (see Eysenck and Derakshan, 2011, for review). It was expected that anxiety of either type (anxious apprehension and anxious arousal) would increase activity in mid-DLPFC associated with self-reported inhibition, activity in posterior DLPFC associated with response inhibition, and ACC activity associated with both measures of inhibition, as these regions have been shown to play prominent roles in attentional control (e.g., Engels et al., 2007, 2010; Banich, 2009). It was also anticipated that anxious apprehension would increase left IFG activity associated with response inhibition, as previous work has shown hyperactivation in this area (Engels et al., 2007).

## Materials and methods

### Participants

Eighty-five paid undergraduate participants (52 females, age *M* = 19.08, *SD* = 1.04) with varying levels of anxiety and depression were recruited from a larger study examining personality, affective, and cognitive risk factors for psychopathology (*N* = 1123; Warren et al., under review; analyses reported here are novel and are orthogonal to Warren et al., under review). From this larger study, participants were selected to be at risk for psychopathology according to their scores on dimensional measures of anxiety and depression (see Psychopathology questionnaires section under Measures). Specifically, participants were selected if they (1) scored at or above the 80th percentile on one of the three psychopathology dimensions and at or below the 50th percentile on the other two dimensions, (2) or if they scored at or above the 80th percentile on all three psychopathology dimensions, or (3) if they scored at or below the 50th percentile on all three psychopathology dimensions. All participants were right-handed, native speakers of English with self-reported normal color vision and hearing, with no neurological disorders or impairments. The Structured Clinical Interview for Axis I Disorders, Non-Patient Edition (First et al., 1997) was administered to all participants. Although participants were not specifically selected based on DSM-IV-TR anxiety or mood disorder diagnosis, approximately 22% met criteria for anxiety disorder only (Anxiety NOS, Generalized Anxiety Disorder, Obsessive Compulsive Disorder, Post Traumatic Stress Disorder, Social Phobia), 9% met criteria for mood disorder only (Major Depressive Disorder or Dysthymia), and 18% met criteria for an anxiety and mood disorder. Participants were given a laboratory tour, informed of the procedures of the study, and screened for claustrophobia and other contraindications for MRI participation. The study was approved by the University of Illinois at Urbana-Champaign Institutional Review Board. Participants were excluded if they had ever experienced loss of consciousness ≥10 min or exhibited current substance abuse or dependence, mania, or psychosis. Additional exclusion criteria included excessive motion or scanner artifact (*n* = 8), signal loss due to substantial uncorrected magnetic susceptibility in areas of interest (*n* = 1), or Stroop reaction time errors greater than 3 standard deviations from the sample mean (*n* = 1).

### Measures

#### Inhibition in everyday life

The Behavior Rating Inventory of Executive Function (BRIEF; self-report version; Guy et al., 2004) is an ecologically sensitive, self-report questionnaire that measures several aspects of EF in an individual's everyday life, including inhibition. Through a series of item-level factor analyses using the BRIEF Warren et al. (under review), identified inhibition, shifting, and updating latent factors consistent with Miyake et al.'s (2000) EF framework. For the present study, the inhibition-item weights (λs; *N* = 1123) identified in Warren et al. (under review) were used to compute participants' behavioral inhibition in everyday life scores. The BRIEF inhibition factor score indexes an individual's ability to resist impulsive responses by pre-empting or stopping one's behavior at the appropriate time and the tendency to act prematurely without foresight in social contexts (Guy et al., 2004). Elevated scores represent impaired cognitive control, manifesting behaviorally as disinhibition and impulsivity.

#### Inhibition in the laboratory

The color-word Stroop task was used as a measure of prepotent response inhibition. Participants completed the color-word Stroop task (Stroop, 1935) during fMRI data acquisition (see below) in which they were asked to press a button indicating the color of the ink in which color words and neutral words were printed, ignoring the dominant tendency to read the words. During the incongruent condition of the Stroop task, cognitive interference is created by the actual meaning of the presented word relative to the ink color in which it is presented (e.g., “RED” in blue ink).

Average RT for correct-response trials was computed for incongruent (e.g., “RED” in blue ink) and neutral trials (e.g., “LOT” in red ink). RT interference scores were computed by subtracting each participant's average neutral RT from their average incongruent RT, divided by their sum ([incongruent RT minus neutral RT]/[incongruent RT plus neutral RT]), and converted to *z* scores across all subjects. Higher interference scores indicated that participants took longer to respond to the ink color of incongruent than of neutral words. No-response trials were excluded from behavioral analyses.

#### Psychopathology questionnaires

Dimensional measures of anxiety and depression, the Penn State Worry Questionnaire (PSWQ; Molina and Borkovec, 1994) and the Anxious Arousal and Anhedonic Depression scales of the Mood and Anxiety Symptom Questionnaire (MASQ; Watson et al., 1995b), were administered during the participant's first visit to the laboratory (see Table 1). Anxious apprehension was measured using the 16-item PSWQ (e.g., “My worries overwhelm me”). Anxious arousal was measured using the relevant 17-item subscale of the MASQ (MASQAA; e.g., “startled easily”). Anhedonic depression was measured using an 8-item subscale from the MASQ (MASQAD8; e.g., “Felt like nothing was very enjoyable”), as it has been shown to predict current and lifetime depressive disorders (Bredemeier et al., 2010). Past research has shown that these measures have good test-retest reliability and good convergent and discriminant validity in undergraduate and clinical samples (Watson et al., 1995a,b; Nitschke et al., 2001).

**Table 1:** **Descriptive statistics**.

	**M**	***SD***	**Min**	**Max**
**QUESTIONNAIRE**				
1. PSWQ (Anxious apprehension)	49.08	18.03	17	80
2. MASQAA (Anxious arousal)	27.56	7.58	17	48
3. MASQAD8 (Anhedonic depression)	16.89	5.77	8	33
**INHIBITION MEASURE**				
1. BRIEF factor score	9.18	2.09	6.32	15.82
2. RT interference	0.11	0.60	−0.30	0.23

N = 85. PSWQ, Penn State Worry Questionnaire. MASQAA, Mood and Anxiety Symptom Questionnaire Anxious Arousal scale. MASQAD8, Mood and Anxiety Symptom Questionnaire Anhedonic Depression 8-item subscale. RT Interference computed by ([incongruent RT minus neutral RT]/[incongruent RT plus neutral RT]).

### Experimental task and stimuli

#### Color-word stroop task

Participants completed color-word and emotion-word Stroop tasks during an fMRI session, and also completed an EEG procedure and a diagnostic interview in other sessions. Only findings from the color-word Stroop task during fMRI are presented here. Hemodynamic data from this same task for an overlapping set of participants was used in a separate study addressing an entirely different research question (Spielberg et al., 2011). The order of presentation of the two tasks within the fMRI session was counterbalanced. The color-word Stroop task consisted of blocks of color-congruent or color-incongruent words alternating with blocks of neutral words. Half of the trials in the congruent and incongruent blocks were neutral to prevent the development of word-reading strategies. This type of blocked-design color-word Stroop task has been shown to effectively elicit Stroop interference (Banich et al., 2000; Milham and Banich, 2005). There were eight orders of stimulus presentation blocks that were counterbalanced across subjects (each participant received one out of eight possible orders). In addition to the word blocks, there were four fixation blocks (one at the beginning, one at the end, and two in the middle of the session) and five rest blocks (one at the beginning, one at the end, and one between each word block). In the fixation condition, a fixation cross intensified in place of word presentation, and in the rest condition the subject was instructed to rest and keep their eyes open while the screen was blank.

Each trial consisted of one word presented in one of four ink colors (red, yellow, green, blue) on a black background, with each color occurring equally often with each word type. The task consisted of congruent trials in which the word named the ink color in which it was printed (e.g., the word “RED” printed in red ink), incongruent trials in which the word named a color incongruent with the ink color in which it was printed (e.g., “GREEN” printed in red ink), and neutral trials in which the word was unrelated to color (e.g., “LOT” in red ink). Neutral words were matched with color words on word frequency and length. Participants responded to the color of the ink with their middle and index fingers using left- and right-hand response boxes.

Participants received 256 trials presented in 16 blocks (four congruent, four incongruent, and eight neutral) of 16 trials each, with a variable ITI (±225 ms) averaging 2000 ms between trial onsets. A trial began with the presentation of a word for 1500 ms, followed by a fixation cross for an average of 500 ms. There was a brief rest period after every fourth block. Additionally, there were four fixation blocks (one at the beginning, one at the end, and two in the middle) in which a brighter fixation cross was presented for 1500 ms. Participants completed 32 practice trials during a low-resolution anatomical scan. No participants failed to understand the task instructions or the mapping between colors and buttons after completing practice trials. Stimuli, word presentation, and reaction-time measurement were controlled by STIM software (James Long Company, Caroga Lake, NY).

#### Image acquisition

A series of 370 fMRI images (16 images per block of 16 stimuli plus rest and fixation periods) were acquired using a gradient-echo echo-planar pulse sequence (TR 2000 ms, TE 25 ms, flip angle 80°, FOV = 22 cm) on a 3T Siemens Allegra head-only scanner. Thirty-eight contiguous oblique axial slices (slice thickness 3 mm, in-plane resolution 3.4375 × 3.4375 mm^2^, 0.3 mm gap between slices) were acquired parallel to the anterior and posterior commissures. After the EPI sequence, a 160-slice MPRAGE structural sequence was acquired (slice thickness 1 mm, in-plane resolution 1 × 1 mm) for registering each participant's functional data to standard space. Prior to the EPI sequence, standard Siemens magnetic field maps were collected with the same slice prescription as the functional scans using a multi-echo gradient echo acquisition (TE's of 10 and 12.46 ms). This field map was used for correction of geometric distortions in the EPI data caused by magnetic field inhomogeneity.

#### fMRI data reduction and analysis

Functional image processing and analysis relied on tools from the FSL analysis package (e.g., MCFLIRT, PRELUDE, FILM, FUGUE, FEAT, FLAME; http://www.fmrib.ox.ac.uk/fsl) and AFNI (http://afni.nimh.nih.gov/afni/). Additional region-of-interest (ROI) analyses were carried out using locally written Matlab programs (e.g., Herrington et al., 2005) and IBM SPSS Statistics version 19.0.

Functional data for each participant were motion-corrected using rigid-body registration, implemented in FSL's linear registration tool, MCFLIRT (Jenkinson et al., 2002). Temporal low-pass filtering was carried out using AFNI's 3dDespike tool (http://afni.nimh.nih.gov/) to remove intensity spikes. The ends of two participants' time series were truncated due to excessive motion. All other participants demonstrated less than 3.3 mm absolute motion or 2 mm relative motion. After motion correction and temporal low-pass filtering, each time series was corrected for geometric distortions caused by magnetic field inhomogeneity (Jezzard and Balaban, 1995; Jenkinson, 2004). Remaining preprocessing steps, single-subject statistics, and group statistics were completed with FEAT. The first three volumes of each participant's functional data were discarded to allow the MR signal to reach a steady state. Each time series was temporally filtered with a nonlinear high-pass filter to attenuate frequencies below 1/212 Hz (to remove drift in signal intensity), mean-based intensity-normalized by the same single scaling factor, and spatially smoothed using a 3D Gaussian kernel (FWHM 5 mm) prior to analysis.

Blood-oxygen-level-dependent (BOLD) activity during the color-word Stroop task was assessed using FILM (FMRIB's Improved Linear Model). Statistical maps were generated via multiple regression on each intracerebral voxel (Woolrich et al., 2001). An explanatory variable (EV) was created for each trial type (congruent, neutral, incongruent, and rest; fixation condition left unmodeled) and convolved with a gamma function to better approximate the temporal course of the BOLD hemodynamic response function (e.g., Aguirre et al., 1998). The contrast of particular interest for this study is the incongruent versus neutral contrast, because incongruent trial performance requires executive function to exert top-down control and resolve conflict. Each EV (i.e., regressor) yielded a per-voxel effect-size parameter estimate (ß) map representing the magnitude of activity associated with that EV for a given participant. Functional activation maps for each contrast were transformed into MNI stereotactic space (ICBM152 2009a Nonlinear Symmetric, 1 × 1 × 1 mm T1 Atlas; Fonov et al., 2009) using FMRIB's Non-Linear Image Registration Tool, FNIRT (Andersson et al., 2007).

Group inferential statistical analyses were carried out using FLAME and SPSS. To identify ROIs for subsequent analysis, activated voxels were identified for the incongruent vs. neutral contrast via two-tailed, per-voxel *t*-tests on contrast β maps converted to z-scores. Monte Carlo simulations via AFNI's AlphaSim program estimated the overall significance level (probability of a false detection) for thresholding these 3D functional z-map images (Ward, 2000). These simulations used a gray-matter mask to limit the number of voxels under consideration (2340 mm^3^) and provided a cluster size (390) and z-value (*z* = 2.97; corresponding *p*-value = 0.003) combination to use for thresholding, resulting in an overall family-wise error rate of 0.05. In order to explore brain regions uniquely associated with inhibition-related constructs, BRIEF inhibition factor score and RT interference (each converted to *z* scores) for each participant were entered as predictors in whole-brain, per voxel, cross-subject regression analyses in FSL. Updating and shifting factor scores (Warren et al., under review) were entered as covariates in order to isolate the specific effects of inhibition. Although there is empirical support for moderate correlations and some overlap among some aspects of EF (Warren et al., under review), inhibition, updating, and shifting components are also behaviorally, genetically, and neurally dissociable (e.g., Miyake et al., 2000; Collette et al., 2005; Friedman et al., 2008; Warren et al., under review). Thus, in order to isolate the specific effects of inhibition in everyday life and the type of inhibition typically observed in the laboratory, brain activity showing distinct relationships with BRIEF inhibition and RT interference was examined by including all EF measures (BRIEF inhibition factor score, RT interference, updating and shifting factor scores) simultaneously in one regression model. This regression analysis produced a β map corresponding to the unique variance associated with each inhibition construct.

Clusters associated with inhibition in everyday life and RT interference that surpassed statistical thresholding were identified as ROIs. To assess the potential effect of psychopathology on neural activity related to these specific inhibition processes, a score for each ROI identified in which BRIEF inhibition factor score and RT interference predicted fMRI was created by averaging β values across voxels in each ROI, for each participant. ROI scores were then entered as the dependent variable in three separate hierarchical linear regressions: (1) PSWQ, MASQAA, and MASQAD8 were entered together as independent variables, (2) their two-way interactions were added together, and (3) their three-way interaction was added. In order to illustrate the resulting moderating effects of psychopathology on ROIs, interactions were plotted and simple slopes tested whether the relationship between brain activity and psychopathology was significantly different from zero at different combinations of high and low levels of anxiety types (see Engels et al., 2010, for details of a similar approach). In Figures 2–4, the relationship between brain activity and anxious apprehension was plotted at high and low levels of anxious arousal. In Figure 5, the relationship between brain activity and depression was plotted at high and low levels of anxious apprehension. For all figures that plot interactions, “high” and “low” refer to ±1 *SD*.

## Results

### Behavioral data

All participants demonstrated color-choice accuracy of at least 85%. As a manipulation check, we examined RT interference for color-word trials. As expected, participants demonstrated more RT interference for incongruent-word trials (*M* = 814 ms, *SD* = 160 ms) than for congruent-word trials (*M* = 633 ms, *SD* = 103 ms), *t*_(84)_ = 15.3, *p* < 0.001, and neutral-word trials (*M* = 652 ms, *SD* = 103 ms), *t*_(84)_ = 15.2, *p* < 0.001.

Descriptive statistics for all of the measures are presented in Table 1, and zero-order correlations among psychopathology and inhibition measures are presented in Table 2,^1^.

**Table 2:** **Zero-order correlations among psychopathology and inhibition-related measures**.

**Measure**	**1**	**2**	**3**	**4**
1. PSWQ (Anxious apprehension)	–			
2. MASQAA (Anxious arousal)	0.48^**^	–		
3. MASQAD8 (Anhedonic depression)	0.49^**^	0.51^**^	–	
4. BRIEF inhibition factor score	0.10	0.35^**^	0.29^**^	–
5. RT interference	0.12	0.13	0.11	0.13

** p ≤ 0.01 (two-tailed).

### fMRI data

#### Brain regions uniquely associated with BRIEF inhibition

**Figure 1:**
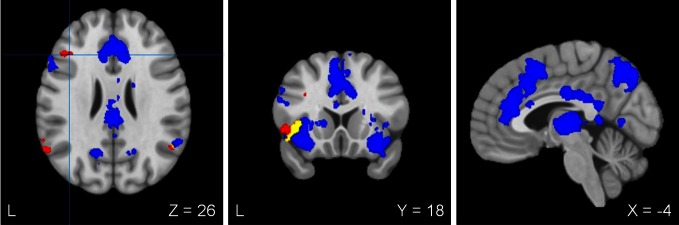
**Areas of activation uniquely associated with either self-reported inhibition in everyday life or prepotent response inhibition.** Red, increased brain activation associated with behavioral inhibition as measured by BRIEF inhibition factor score. Blue, decreased brain activation associated with prepotent response inhibition as measured by RT interference. Yellow, brain activation overlap between BRIEF inhibition factor score and RT interference. L, Left. Location of crosshairs emphasizes a differentiation of mid-DLPFC (red) and posterior DLPFC (blue) regions.

Table 3 lists seven regions that were positively correlated with the BRIEF inhibition factor score. In line with hypotheses, higher BRIEF inhibition factor scores were associated with more activation in left mid-DLPFC (middle frontal gyrus; see Figure 1) and left IFG, regions that are generally associated with implementing inhibition-related processes. Additional clusters emerged in frontal pole, OFC, and supramarginal and angular gyrus regions. There were no significant clusters negatively correlated with BRIEF inhibition factor scores.

**Table 3:** **Distinct effects of brief inhibition factor score**.

**Region**	**Cluster size**	**Mean Z**	**COM Location**			**Max Z Location**		
			***x***	***y***	***z***	***x***	***y***	***z***
**INCONGRUENT VERSUS NEUTRAL WORDS^a^**								
L frontal pole, OFC	397	3.30	−46	39	−16	−48	40	−17
L inferior frontal gyrus (IFG), anterior insula	1346	3.25	−46	16	0	−51	17	−2
L frontal pole, IFG-pars triangularis	423	3.35	−47	39	6	−46	40	6
R lateral occipital cortex, angular gyrus, TPJ	498	3.18	53	−59	21	53	−60	20
L middle frontal gyrus (mid-DLPFC)	402	3.19	−40	26	28	−43	25	27
L supramarginal gyrus	4851	3.26	−54	−53	41	−54	−44	52
R angular gyrus, lateral occipital cortex	558	3.31	48	−55	54	50	−56	54

N = 85. COM, center of mass; R, right; L, left; DLPFC, dorsolateral prefrontal cortex; OFC, orbitofrontal cortex; TPJ, temporoparietal junction.

a z-scores > 2.9677, cluster-size ≥ 390 (corrected p < 0.05).

#### Moderation of brain activity by psychopathology associated with BRIEF inhibition factor score

No significant moderation by anxiety, depression, or their interactions emerged with any of the self-reported inhibition ROIs.

#### Brain regions uniquely associated with RT interference

Table 4 lists a network of regions that were negatively correlated with RT interference. In line with hypotheses, higher RT interference was associated with less activation in left posterior DLPFC (middle frontal gyrus), bilateral IFG, and ACC (rostral, dorsal, and anterior mid-cingulate), as well as regions that are generally associated with attentional control and motor response coordination (e.g., premotor cortex, frontal eye fields, posterior parietal cortex, precuneus; see Figure 1). Additional clusters emerged in occipital cortex, thalamus and caudate, parahippocampal gyrus, frontal pole, OFC, and supramarginal and angular gyrus regions (see Figure 1). There were no significant clusters positively correlated with RT interference.

**Table 4:** **Distinct effects of RT interference**.

**Region**	**Cluster size**	**Mean Z**	**COM location**			**Max Z location**		
			***x***	***y***	***z***	***x***	***y***	***z***
**Incongruent vs. neutral words^a^**								
Bilateral thalmaus, caudate; LH OFC, insula, IFG	30997	−3.67	−12	−5	5	−6	−21	11
R OFC, insula, IFG	7029	−3.45	36	17	−11	28	17	−16
R temporal occipital fusiform cortex	442	−3.23	37	−47	−21	36	−42	−21
R lingual gyrus	566	−3.31	5	−81	−15	4	−80	−12
L lateral occipital cortex, posterior ITG	4764	−3.32	−38	−77	−11	−46	−62	−8
R temporal occipital fusiform cortex, ITG	1119	−3.25	45	−61	−16	46	−56	−18
L lateral occipital cortex, occipital pole	581	−3.20	33	−89	−10	35	−86	−9
R middle temporal gyrus	1316	−3.44	54	−30	−7	54	−31	−7
R parahippocampal gyrus	549	−3.42	20	−30	−9	22	−28	−8
rACC, dACC, aMCC	19171	−3.49	0	25	32	10	25	24
Bilateral precuneous cortex	14804	−3.54	−7	−67	39	−7	−66	45
R frontal pole	942	−3.40	26	54	13	28	55	9
L middle frontal gyrus (posterior DLPFC)	1980	−3.49	−54	15	32	−53	13	41
R angular gyrus	399	−3.28	58	−52	24	58	−51	23
L supramarginal gyrus	462	−3.16	−52	−41	38	−50	−37	43
L supramarginal gyrus, angular gyrus	491	−3.17	−33	−46	38	−31	−44	36
L middle frontal gyrus (DLPFC), premotor cortex, FEF	1981	−3.40	−26	−2	53	−32	−3	54

N = 85. COM, center of mass; R, right; L, left; DLPFC, dorsolateral prefrontal cortex; OFC, orbitofrontal cortex; IFG, inferior frontal gyrus; ITG, inferior temporal gyrus; dACC, dorsal anterior cingulate cortex; rACC, rostral anterior cingulate cortex; aMCC, anterior mid-cingulate cortex; FEF, frontal eye field.

a z-scores > 2.9677, cluster-size ≥ 390 (corrected p < 0.05).

#### Moderation of brain activity by psychopathology associated with RT interference

**Figure 2:**
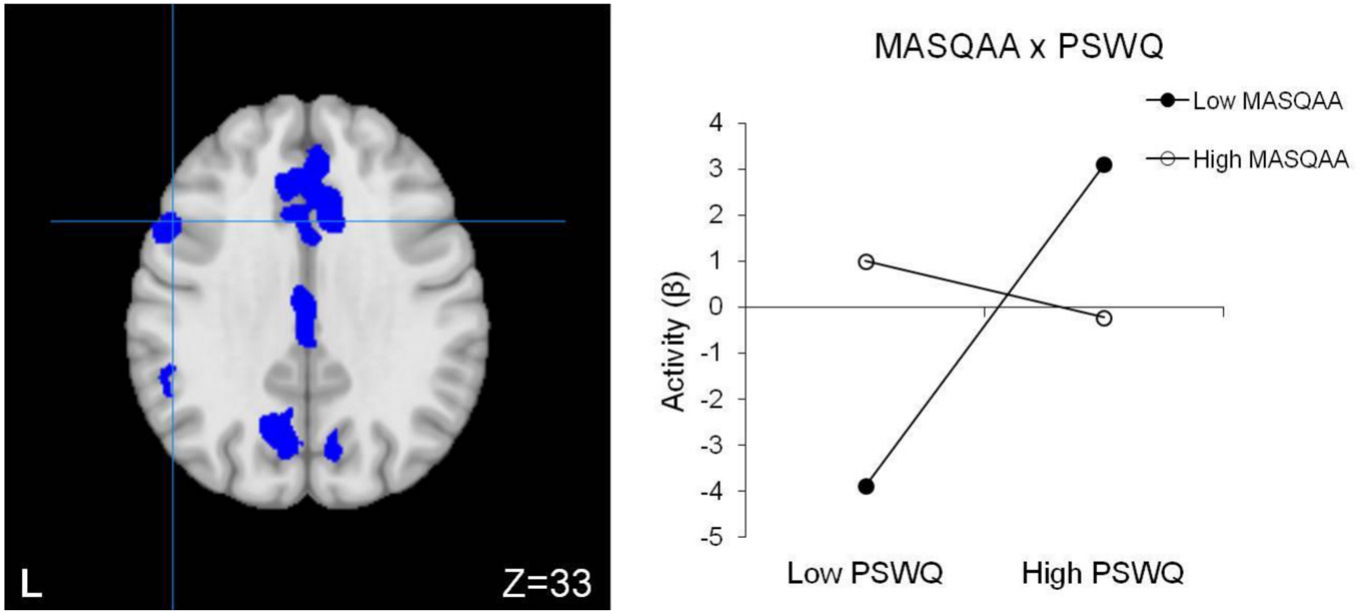
**Posterior DLPFC activation for RT interference.** Blue, decreased brain activation associated with RT interference. L, left. Graphing the MASQAA × PSWQ interaction illustrates that anxious apprehension's relationship with left posterior DLPFC depends on the level of co-occurring anxious arousal.

**Figure 3:**
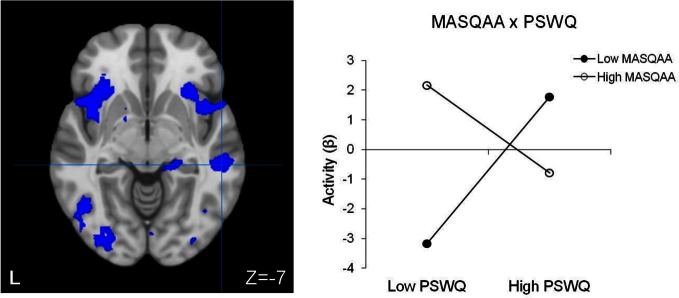
**Right MTG activation for RT interference.** Blue, decreased brain activation associated with RT interference. L, left. Graphing the MASQAA × PSWQ interaction illustrates that anxious apprehension's relationship with right MTG depends on the level of co-occurring anxious arousal.

**Figure 4:**
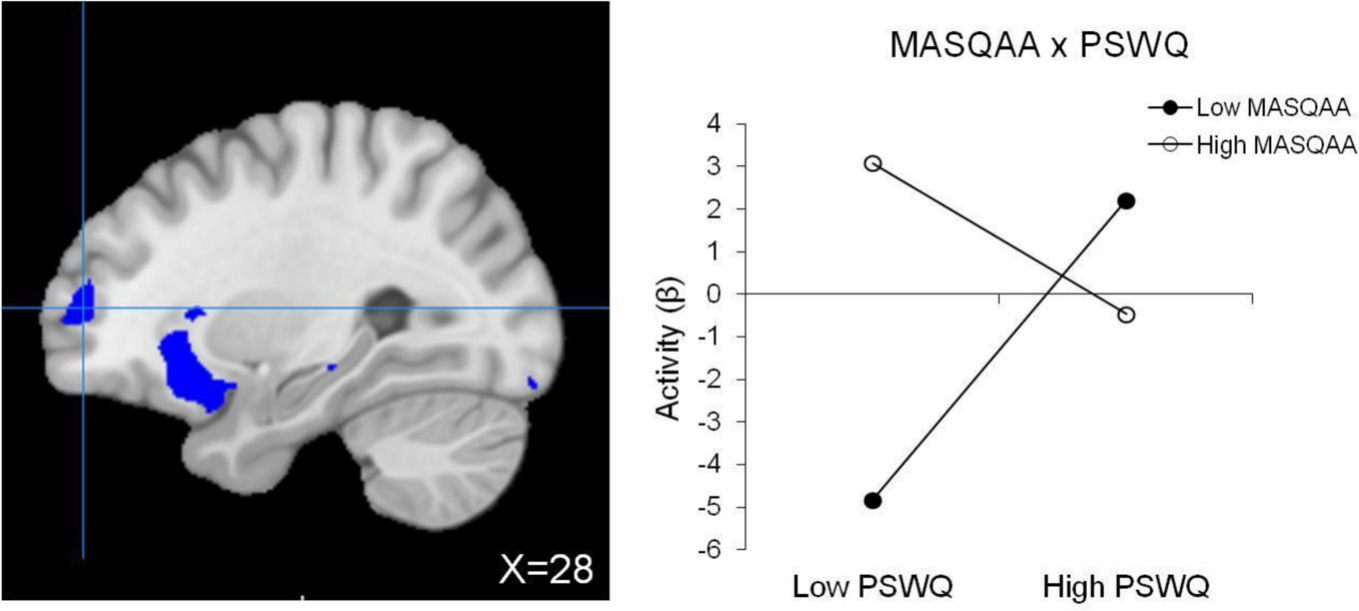
**Right frontal pole activation for RT interference.** Blue, decreased brain activation associated with RT interference. Graphing the MASQAA × PSWQ interaction illustrates that anxious apprehension's relationship with right frontal pole depends on the level of co-occurring anxious arousal.

**Figure 5:**
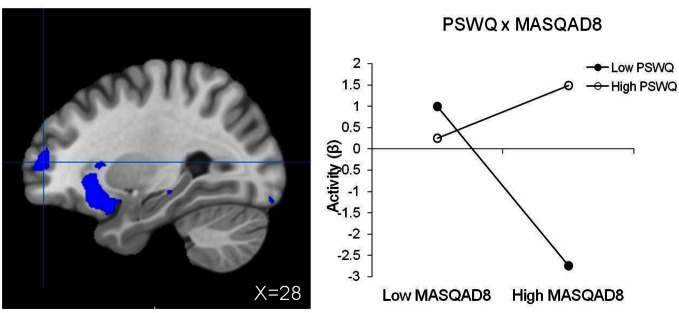
**Right frontal pole activation for RT interference (same region pictured in Figure 4).** Blue, decreased brain activation associated with RT interference. Graphing the PSWQ × MASQAD8 interaction illustrates that anxious apprehension's relationship with right frontal pole depends on the level of co-occurring anhedonic depression.

No significant main effect of anxiety type, depression, or their three-way interaction emerged. Table 5 lists regions with two-way interactive effects for anxiety and depression for response-inhibition-related brain activity. Three regions were moderated by four, two-way interactions. A PSWQ × MASQAA interaction emerged for left posterior DLPFC (Figure 2). As illustrated in Figure 2, increased anxious apprehension was associated with increased left posterior DLPFC activation, but only when anxious arousal was low. Tests of simple slopes showed that this was the only significant slope [*t*_(78)_ = 2.84, *p* < 0.01]. A PSWQ × MASQAA interaction was found for right middle temporal gyrus (MTG; Figure 3). Tests of simple slopes showed that increased anxious apprehension was associated with decreased right MTG activation at high levels of anxious arousal [*t*_(78)_ = −2.86, *p* < 0.01] but with increased activation at low levels of anxious arousal [*t*_(78)_ = 2.02, *p* = 0.05]. Finally, two interactions emerged for right frontal pole (Figures 4 and 5). Similar to right MTG, increased anxious apprehension was associated with decreased right frontal pole activation at high levels of anxious arousal [*t*_(78)_ = −3.47, *p* < 0.001] but with increased activation at low levels of anxious arousal [*t*_(78)_ = 2.91, *p* < 0.01; Figure 4]. Additionally, a PSWQ × MASQAD8 interaction emerged in which high levels of anhedonic depression were associated with decreased right frontal pole activity at low levels of anxious apprehension. Tests of simple slopes showed that this was the only significant slope [*t*_(78)_ = −3.55, *p* < 0.001; Figure 5].

**Table 5:** **Regression analyses for two-way interactive effects of anxiety and depression on RT interference ROIs**.

**Region**		***R*^2^**	**Δ*R*^2^**	**Test**	***p***
L middle frontal gyrus (posterior DLPFC)	PSWQ × MASQAA		0.08	*t*_(78)_ = −2.65	0.01
	Full model	0.156		*F*_(6, 78)_ = 2.40	0.04
R middle temporal gyrus	PSWQ × MASQAA		0.07	*t*_(78)_ = −2.57	0.01
	Full model	0.164		*F*_(6, 78)_ = 2.55	0.03
R frontal pole	PSWQ × MASQAA		0.13	*t*_(78)_ = −3.48	<0.01
	Full model	0.185		*F*_(6, 78)_ = 3.00	0.01
R frontal pole	PSWQ × MASQAD8		0.04	*t*_(78)_ = −2.96	0.05
	Full Model	0.185		*F*_(6, 78)_ = 3.00	0.01

N, 85. PSWQ, Penn State Worry Questionnaire; MASQAA, Mood and Anxiety Symptom Questionnaire Anxious Arousal scale; MASQAD8, Mood and Anxiety Symptom Questionnaire Anhedonic Depression 8-item subscale; R, right; L, left; DLPFC, dorsolateral prefrontal cortex. ΔR^2^is the incremental variance associated with the interaction term, with its individual constituents already in the model.

## Discussion

The present study examined neural mechanisms supporting ecologically sensitive versus laboratory-based measures of inhibitory function in order to clarify the broader construct of inhibition as well as their role in psychopathology. Brain-activation results were consistent with regions of interest predicted to be associated with inhibition-related processes. In general, worse self-reported inhibition in everyday life (elevated BRIEF factor score) was associated with increased activity in brain regions typically associated with inhibitory function (left DLPFC, left IFG, bilateral inferior parietal cortex; Figure 1). In contrast, worse performance on the laboratory task (increased RT interference) was associated with decreased brain activity in these regions as well as ACC (see Figure 1). Importantly, although DLPFC activity was associated with both measures of inhibitory functions, each measure exhibited unique relationships with DLPFC. As predicted, worse self-reported inhibition was associated with increased activity in mid-DLPFC, and greater RT interference was associated with less activity in posterior DLPFC. These differential patterns of inhibition-related processes suggest a distinct role for each DLPFC area.

The cascade of control model (Banich et al., 2000, 2009; Milham and Banich, 2005; Banich, 2009) identifies aspects of EF that are critical for inhibiting responses, including biasing responses toward task-relevant processes, biasing attention toward task-relevant representations, response selection, and response evaluation. This model proposes that distinct areas of DLPFC implement these functions, which are necessary for cognitive control. Posterior DLPFC imposes a top-down attentional set toward task-relevant processes, maintains the overall task goals, and subsequently biases other brain regions (e.g., mid-DLPFC, dorsal ACC, parietal cortex) toward processing task-relevant information. In contrast, mid-DLPFC is involved in selecting and maintaining the most relevant aspects of task stimuli (Banich, 2009) and has been suggested to play an important role in stimulus-driven attentional control (Crocker et al., 2012). Mid-DLPFC is thought to be involved in interrupting top-down processing to reorient attention to stimuli that have been identified as relevant (Corbetta et al., 2008; Crocker et al., 2012) and therefore could be said to be critically involved in tracking and multitasking functions. In the context of present findings, a behavioral manifestation of a high BRIEF inhibition factor score is impulsivity. Thus, mid-DLPFC hyperactivity associated with increased BRIEF inhibition factor score could reflect paying attention to too many task representations and/or hyper-focusing on stimulus properties, which could disrupt relevant task goal maintenance. In line with this interpretation, hyperactivity in mid-DLPFC has been linked to over-engagement with irrelevant features of stimuli (the meaning of threat-related words in an emotion-word Stroop task), interfering with processing task-relevant features (word color; Engels et al., 2010).

In contrast, a negative correlation between RT interference and posterior DLPFC was observed, such that the greater the RT interference, the less the brain activity. Given DLPFC's prominent role in top-down attentional control (Milham et al., 2003), if posterior DLPFC fails to impose a top-down attentional set toward task-relevant processes (inferred by decreased activity), one would anticipate greater RT interference. Present results are consistent with other findings (Banich et al., 2000; Milham et al., 2003; Milham and Banich, 2005).

In line with the cascade-of-control model, RT interference was also associated with areas of ACC that are involved in response selection and response evaluation. Specifically, the model asserts that there is a temporal cascade of cognitive operations, such that, following DLPFC activation, dorsal ACC selects the appropriate response among available response options. When incorrect responses are made during a task, more anterior regions of ACC signal posterior DLPFC to assert greater top-down control for task performance, requiring re-initiation of certain steps in the temporal cascade of events. In addition to posterior DLPFC and ACC, present findings for regions of activation for RT interference were consistent with those implicated in a distributed network associated with response inhibition, including bilateral IFG, as well as regions that are generally associated with attentional control and coordinating motor responses (e.g., premotor cortex, frontal eye fields, posterior parietal cortex, precuneus; Corbetta et al., 2008; Banich, 2009).

Contributing to understanding EF deficits in psychopathology, select patterns of brain activation for response inhibition (RT interference) were modulated by anxiety and depression. A two-way interaction emerged for left posterior DLPFC in which greater activity was associated with high anxious apprehension, but only when anxious arousal was low. Anxious apprehension typically involves elaborated verbal processing and worry. Given that posterior DLPFC is involved in imposing top-down attentional control and maintaining task set, greater activity in this area may reflect an attempt to compensate for anxious apprehension (which can be inferred to impair the efficiency of this inhibition-related function). Considerable evidence suggests that anxiety is often associated with increased susceptibility to distraction (see Derakshan and Eysenck, 2009, for review), hypothesized to reflect impaired inhibition (e.g., Eysenck and Derakshan, 2011). According to attentional control theory, anxiety impairs processing *efficiency* to a greater extent than it impairs performance *effectiveness* (i.e., quality of performance; Eysenck et al., 2007) and manifests in greater activation in brain regions associated with attentional control. Present findings suggest that individuals high in anxious apprehension (worry), a specific dimension of anxiety, especially when anxious arousal is low, are more susceptible to distraction and thus to impaired efficiency of inhibition during cognitively demanding tasks (i.e., inhibiting the dominant tendency to read the color word). The fact that anxious apprehension and anxious arousal are not associated with error rate could reflect compensation by posterior DLPFC (inferred from greater activity). Despite its disrupting impact on efficiency, increased activity associated with anxious apprehension or worry may be adaptive under some circumstances. Anxious apprehension ameliorated a depression-related suppression of activity in DLPFC (again, only when anxious arousal was low; Engels et al., 2010). Types of anxiety and depression may thus interact to influence optimal levels of activity in brain regions associated with cognitive control, which in turn may affect the balance of goal maintenance vs. stimulus-driven or contextual processing.

A two-way interaction emerged for right MTG in which greater RT interference activity was associated with high anxious apprehension when anxious arousal was low and with decreased activity when anxious arousal was high. Additional examination of this interaction revealed one significant slope, such that brain activity increased as anxious arousal increased, but only when anxious apprehension was low. Right MTG is thought to interact with a network of regions involved in detecting and responding to threat (e.g., Compton et al., 2003; Corbetta et al., 2008). This region may be a part of a system that functions adaptively to switch between top-down attentional control and more stimulus-driven processing (Corbetta et al., 2008). Using an emotion-word Stroop task, Engels et al. (2007) demonstrated that negative emotion words elicited greater right middle-temporal/inferior-temporal activity in an anxious arousal group. Importantly, present results generalize Engels' et al. (2007, 2010) findings to non-emotional contexts, suggesting that anxiety-modulated increases in activity in this region interfere with an inhibition-related function for cognitive control. Additionally, in a non-overlapping sample, Engels et al. (2010) found that anxious arousal ameliorated depression-related suppression of activity in this region, in response to threatening words. Again, these findings suggest that under some circumstances anxiety-related activation has an adaptive function.

Similar to the pattern observed for right MTG, greater right frontal pole (BA10) activity was associated with high anxious apprehension when anxious arousal was low and with decreased activity when anxious arousal was high. Additionally, anxious apprehension diminished depression-related suppression of activity in this region. Rostral PFC (BA10) has been implicated in supporting a wide range of functions including prospective memory, multitasking, and “mentalizing” or reflecting on mental states (see Burgess et al., 2007, for review). According to the gateway hypothesis (Burgess et al., 2007), rostral PFC is part of a cognitive control system that biases the relative influence of stimulus-independent and stimulus-oriented thought. Lateral regions of rostral PFC are associated with stimulus-independent cognition, the mental processes that accompany self-generated or self-maintained thought that is not provoked by or directed toward an external stimulus (i.e., task-irrelevant thought). The right frontal pole region in the present study overlaps with the lateral area of rostral PFC identified by Burgess et al. (2007) as supporting stimulus-independent function. Anxious apprehension modulation of brain activity in this region (when anxious arousal is low) could reflect task-irrelevant thoughts such as worry, an example of stimulus-independent cognition, potentially interfering with task efficiency. However, anxious apprehension also interacted with depression in this same region, such that depression-related hypoactivity decreased as anxious apprehension increased. Findings suggest that whereas anxious apprehension could interfere with task efficiency when anxious arousal is low, worry could potentially be adaptive for task performance at high levels of depression.

Contrary to hypotheses, no significant moderation of anxiety, depression, or their interactions emerged with any of the ROIs associated with self-reported inhibition in everyday life. It is possible that the color-word Stroop task does not robustly engage inhibition-related neural mechanisms that implement the kind of everyday inhibitory control that is affected by anxiety or depression. Another possible explanation for the lack of significant findings is the general nature and range of everyday scenarios that the self-reported inhibition score indexes. Although the self-reported inhibition score may be sensitive to neural mechanisms supporting this function, the measure may not be specific enough to capture anxiety and depression deficits.

Maintenance of top-down attentional control is typically assumed to be the main function of DLPFC. However, present results suggest a more nuanced view of DLPFC in the context of cognitive control, as sub-regions were differentiated by two aspects of inhibition-related functions. Present results support an emerging view that areas within DLPFC (mid and posterior) may provide distinct contributions to cognitive control (Banich, 2009). Whereas mid-DLPFC has been associated with stimulus-driven attentional control (Crocker et al., 2012), posterior DLPFC imposes a top-down attentional set that maintains overall task goals. In combination, these regions are involved in preventing irrelevant information from entering working memory. In the context of the current study, present findings suggest that differing inhibition-related mechanisms may contribute to the efficiency in which information is maintained in working memory, as well as resistance to interference.

DLPFC dysfunction has been implicated as a source of cognitive impairment in a range of psychopathology, including depression and anxiety (Engels et al., 2007, 2010; Levin et al., 2007; Warren et al., 2008; Herrington et al., 2010; Silton et al., 2010). Although inhibitory functions are not the only factors that are associated with cognitive dysfunction in psychopathology, their differing neural mechanisms certainly have probative value. For example, theories of depression (Joormann et al., 2007) and anxiety (Eysenck et al., 2007) postulate inhibitory dysfunction as a source of symptom development and maintenance, although specific inhibitory functions are not addressed. Indeed, present findings demonstrate that only response inhibition-related brain activity (RT interference) was significantly moderated by psychopathology. Thus, assessing individual differences in specific inhibition-related functions and their neural mechanisms might be a profitable approach to understanding how “inhibition” contributes to cognitive and emotional disruptions in psychopathology.

Anxiety-modulated hyperactivity in brain regions associated with cognitive control suggests a vulnerability to distraction, even in conditions when there is no manipulated threat (e.g., color-word Stroop task). In the same vein, Silton et al. (2011) found that, as anxious apprehension increased, increased dACC activity (another key region associated with implementing cognitive control) was associated with greater Stroop interference (less efficient performance). Neuroimaging evidence and theories of anxiety (e.g., Eysenck et al., 2007; Eysenck and Derakshan, 2011) suggest that excessive anxiety manifests as hyperactivity in brain regions associated with attentional control during task performance, a pattern of activity that is thought to reflect compensation. However, there are limits to compensation, and it is important to determine when compensation may break down, such as when individuals with excessive anxiety are under stress. Under such conditions, it is likely that functional impairments become overtly apparent in the contexts in which they are most detrimental (e.g. during an exam or meeting an important deadline).

Present findings reveal specific inhibition-related neural mechanisms associated with PFC, particularly sub-regions of DLPFC, and MTG, as well as the modulating effects of specific combinations of anxious apprehension, anxious arousal, and anhedonic depression. Although these effects indicate potential sources of impaired or disrupted performance, under some circumstances they may function to ameliorate or compensate for imbalances in optimal levels of activity in systems of cognitive control.

### Conflict of interest statement

The authors declare that the research was conducted in the absence of any commercial or financial relationships that could be construed as a potential conflict of interest.

## Footnotes

^1^Given the significant zero-order correlations among the BRIEF Inhibition Factor Score and psychopathology questionnaires, there is a potential for selection bias with respect to brain activity in regions of interest. However, given that the psychopathology measures did not significantly moderate the relationship between brain activity and BRIEF Factor Score, the potential for non-independence is not an issue.
